# Eco-friendly synthesis of copper nanoparticles by using *Ralstonia* sp. and their antibacterial, anti-biofilm, and antivirulence activities

**DOI:** 10.1016/j.bbrep.2025.101978

**Published:** 2025-03-13

**Authors:** Narges Vakili, Morahem Ashengroph, Aram Sharifi, Musa Moetasam Zorab

**Affiliations:** a Department of Biological Science, Faculty of Science, University of Kurdistan, P.O. Box 416, Sanandaj, Kurdistan, Iran; b Research Center for Nanotechnology, University of Kurdistan, P.O. Box 416, Sanandaj, Kurdistan, Iran; c Department of Animal Science, Faculty of Agriculture, University of Kurdistan, Sanandaj, Kurdistan, Iran; d Department of Physics, College of science, University of Halabja, Kurdistan region, Iraq

**Keywords:** Green method, Copper nanoparticles, Antimicrobial, Anti-biofilm, Antivirulence

## Abstract

Biosynthesized nanoparticles (NPs) created through environmentally friendly and low-toxicity methods show great potential for various nanotechnology applications. In particular, copper nanoparticles (Cu-NPs) are promising for medical uses. This study aims to explore the eco-friendly synthesis of Cu-NPs and their potential as a novel strategy to combat antimicrobial resistance. Cu-NPs were synthesized using *Ralstonia* sp. KF264453 and characterized with techniques including ultraviolet–visible (UV–Vis) spectroscopy, field emission scanning electron microscopy (FESEM), energy dispersive X-ray spectroscopy (EDX), dynamic light scattering (DLS), zeta potential analysis, X-ray diffraction (XRD), and Fourier transform infrared spectroscopy (FT-IR). The antibacterial properties of the NPs and their synergistic effects with common antibiotics were assessed. The study also investigated their impact on bacterial cell membrane disruption, biofilm formation, efflux pump activity, and motility. UV–Vis analysis indicated a significant absorption peak at 552 nm, confirming surface plasmon resonance (SPR) for Cu-NPs. FESEM images revealed predominantly spherical NPs with an average size of 69.7 nm. DLS measurements indicated a hydrodynamic diameter of 78.2 nm due to stabilizing biomolecules. A zeta potential of −5.1 mV suggested moderate colloidal stability, suitable for short-term biomedical applications. XRD analysis confirmed a face-centered cubic (FCC) crystalline structure with an average crystallite size of 45 nm. FT-IR spectra detected functional groups, indicating that proteins, carbohydrates, lipids, and amino acids may have contributed to the synthesis and stabilization of the NPs. Cu-NPs showed notable antibacterial efficacy, with minimum inhibitory concentrations (MIC) between 0.625 and 5 μg/mL and minimum bactericidal concentrations (MBC) ranging from 5 to 20 μg/mL. They improved the effectiveness of penicillin and cefixime, enhanced membrane permeability, inhibited biofilm formation, disrupted efflux pump activity in *Staphylococcus aureus* SA-1199B, and decreased swarming motility in *Pseudomonas aeruginosa*. Cu-NPs demonstrate strong antimicrobial activity, inhibit biofilm formation and efflux pump function, and enhance the effectiveness of conventional antibiotics. While they show promise in combating antimicrobial resistance, further research is needed to assess their clinical potential and safety for medical use.

## Highlights

• Cu-NPs were sustainably synthesized using the eco-friendly *Ralstonia* sp. KF264453.
• Cu-NPs exhibited notable antibacterial activity, with a MIC range of 0.625–5 μg/mL.
• Cu-NPs enhanced the effectiveness of penicillin and cefixime against antibiotic-resistant strains.
• Cu-NPs disrupted microbial membranes and inhibited biofilm formation as antibacterial mechanisms.
• Cu-NPs represent innovative antimicrobial agents in the fight against antibiotic resistance.

## Graphical abstract

**Figure:**
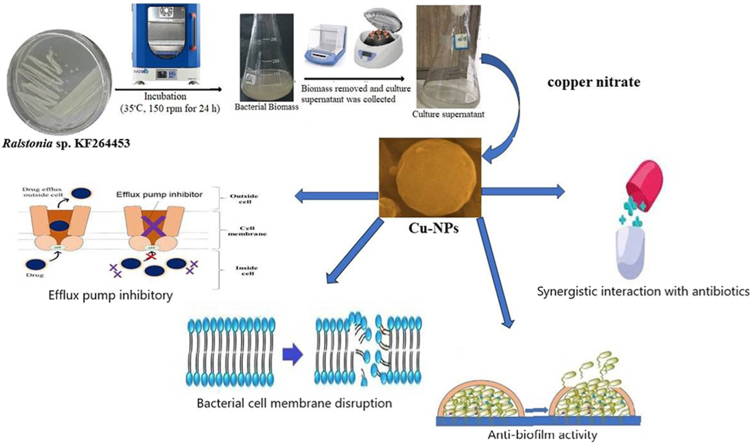

## 1 Introduction

Nanotechnology is rapidly transforming medicine, especially in the development of NPs [1]. These nanoscale materials, measuring 1–100 nm, have unique properties that improve their efficacy in medical applications, such as treating bacterial infections [2]. NPs interact with microbial cells at the nanoscale, disrupting essential cellular functions and providing innovative strategies against antibiotic-resistant pathogens [3]. Their small size allows them to penetrate bacterial membranes, destabilize structures, and disrupt vital metabolic processes. Some NPs generate reactive oxygen species (ROS) that induce oxidative stress, damaging bacterial components [[3], [4], [5]]. Additionally, certain NPs reduce bacterial virulence by interfering with communication pathways and infection mechanisms, effectively targeting resistant strains while minimizing resistance development—a notable advantage over traditional antibiotics [6]. Cu-NPs are gaining interest for their cost-effective, versatile, and broad-spectrum antimicrobial properties, making them a viable alternative to precious metals such as silver and gold [7,8]. Their superior antibacterial efficacy over bulk copper is due to a high surface area-to-volume ratio, which increases interaction with microbial cells and enhances biofilm inhibition [8]. Cu-NPs fight bacteria by producing ROS and releasing copper ions, which damage bacterial membranes and disrupt vital cellular functions [8]. Traditional nanoparticle synthesis typically relies on hazardous chemicals and produces toxic by-products, which pose environmental and safety risks. In contrast, green synthesis utilizes biological resources such as plants, bacteria, and fungi to create NPs with lower environmental impact and improved biocompatibility through biomolecular coatings [5,9].

Biological methods have effectively synthesized metal-based NPs with diverse applications. Zinc oxide nanoparticles (ZnO-NPs) are valued for their photocatalytic and antioxidant properties in environmental and biomedical fields [10]. Zirconium nanoparticles (Zr-NPs) show strong antibacterial capabilities, making them promising for fighting microbial infections [11]. Tellurium nanoparticles (Te-NPs) possess antioxidant, antibacterial, antifungal, and cytotoxic properties, enabling a range of therapeutic and industrial uses [12]. Additionally, selenium nanoparticles (Se-NPs) have gained significant attention for their anticancer properties and effectiveness as photothermal agents in cancer therapy, offering innovative solutions for targeted cancer treatments [13].

Bacteria are particularly effective, utilizing their metabolic by-products to reduce metal ions and accumulate metals intracellularly. This process is pivotal for combating antimicrobial resistance (AMR) by enabling the efficient creation of new antibacterial agents [14,15]. This study investigates the eco-friendly synthesis of Cu-NPs using *Ralstonia* sp., highlighting their antimicrobial, biofilm-inhibiting, and virulence-reducing properties against major pathogens. The findings present a sustainable approach with significant therapeutic potential, positioning these Cu-NPs as a promising tool against antimicrobial resistance. This research advances applications in public health and nanotechnology, addressing bacterial infections and AMR.

## 2 Materials and methods

### 2.1 Bacterial strains and culture conditions

This study utilized the bacterial strain *Ralstonia* sp. KF264453, isolated from soil near the Sarcheshmeh copper mine in Kerman, for synthesizing Cu-NPs [16]. Antibacterial assays were conducted against *S. aureus* ATCC 25923, *S. aureus* SA1199B (a *norA*-overexpressing strain), *Bacillus cereus* ATCC 11778, *Escherichia coli* ATCC 25922, and *P. aeruginosa* ATCC 27853, sourced from the Persian Type Culture Collection (PTCC) in Iran. The cultures were grown on Merck media (Darmstadt, Germany) under aerobic conditions at 37 °C for 24–36 h.

### 2.2 Green synthesis of Cu-NPs

Cu-NPs were green-synthesized using *Ralstonia* sp. KF264453. Biomolecules in the bacterial supernatant, such as enzymes and metabolites, served as natural reducing and stabilizing agents to convert Cu^2+^ ions into metallic Cu^0^. This eco-friendly method avoids the toxic chemicals used in conventional synthesis. The copper nitrate concentration (1–4 mM) was optimized for efficient nanoparticle formation. A high-purity copper (II) nitrate (Cu(NO_3_)_2_·3H_2_O) stock solution (1–4 mM) was prepared in sterile deionized water, filtered through a 0.22 μm membrane, and stored at 4 °C. The bacterial culture was grown overnight in sterile nutrient broth (5 g peptone and 3 g meat extract per liter) at 35 °C with shaking at 150 rpm. After incubation, the cultures were centrifuged at 5000×*g* for 10 min, and the supernatant was collected as the bacterial extract. For nanoparticle synthesis, 50 mL of the Cu(NO_3_)_2_ solution (1–4 mM) was mixed with 50 mL of the bacterial supernatant and incubated at room temperature for 24 h with shaking at 150 rpm. The mixture was then purified by centrifugation at 10,000×*g* for 30 min. The resulting pellets were washed three times with sterile deionized water to remove residual chemicals. Finally, the samples were freeze-dried (Alpha 1-2Dplus, Christ, Germany) for 12 h to prepare for further analysis.

### 2.3 Characterization of Cu-NPs

Green-synthesized Cu-NPs were characterized through various spectroscopic and microscopic techniques to confirm their structural, morphological, and chemical properties. UV–Vis spectroscopy (Specord 210 Plus, Analytik Jena, Germany) identified the surface plasmon resonance (SPR) peak, indicating nanoparticle formation [17]. Morphological traits, including size, shape, and elemental composition, were assessed using FESEM-EDX (MIRA3, TESCAN, Czech Republic), and analyzed via ImageJ software. Dynamic light scattering (DLS) and zeta potential analysis (Malvern, UK) determined the hydrodynamic size distribution, surface charge, and colloidal stability of Cu-NPs in aqueous solutions. FTIR spectroscopy (Bruker, USA) detected surface functional groups, likely derived from bacterial supernatant biomolecules acting as capping agents. X-ray diffraction (XRD) analysis using a Philips PW1730 with CuKα radiation (λ = 1.540598 Å) over a 2θ range of 5°–80° confirmed the crystalline structure of the Cu-NPs. The average crystallite size was determined using the Debye–Scherrer equation, offering insights into their nanoscale dimensions [18].

### 2.4 Antibacterial properties of Cu-NPs

The minimum inhibitory concentration (MIC) of Cu-NPs was assessed using the broth microdilution method in a 96-well microplate with Mueller-Hinton broth (MHB). MIC values varied from 0.312 to 160 μg/mL. Bacterial suspensions, adjusted to 0.5 McFarland and diluted in MHB, were added to each well, achieving a final concentration of 4–5 × 10^5^ CFU/mL. After overnight incubation at 37 °C, the MIC was determined as the lowest Cu-NP concentration that inhibited visible bacterial growth. The minimum bactericidal concentration (MBC) was established by transferring 10 μL samples from wells without growth onto Mueller-Hinton agar (MHA) for further evaluation. After overnight incubation at 37 °C, colony counts verified bacterial viability, with the MBC identified as the lowest Cu-NP concentration causing a 99.9 % bacterial reduction. Vancomycin and gentamicin were used as controls for Gram-positive and Gram-negative bacteria, respectively [19].

### 2.5 Synergistic interaction between Cu-NPs and penicillin or cefixime

**Fig. 1:**
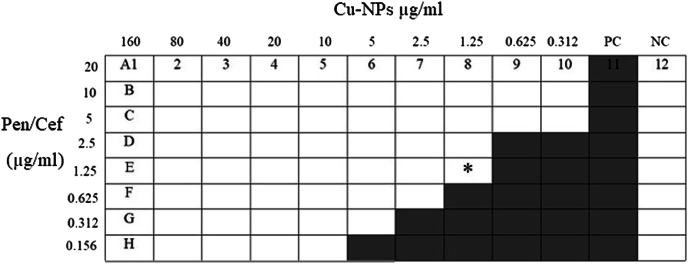
Schematic of the checkerboard method illustrating the synergy between Cu-NPs and antibiotics. Shaded cells denote bacterial growth, while unshaded cells indicate no growth. PC: positive control; NC: negative control; Pen: penicillin; Cef: cefixime; ∗: MIC of combined substances.

To evaluate the synergistic effects of Cu-NPs with penicillin and cefixime, we conducted a checkerboard assay to determine the Fractional Inhibitory Concentration (FIC) Index. We prepared twofold serial dilutions of Cu-NPs, ranging from 0.312 to 160 μg/ml, in the rows of a 96-well microtiter plate and cross-diluted them with twofold serial dilutions of penicillin (Merck, Darmstadt, Germany) for Gram-positive bacteria and cefixime (Merck, Darmstadt, Germany) for Gram-negative bacteria (Fig. 1). Antibiotic concentrations ranged from 0.156 to 20 μg/mL. Bacterial strains were inoculated on the plates and incubated for 24 h at 37 °C. MIC values for the combinations were determined as the lowest concentrations that fully inhibited bacterial growth. The FIC index is calculated by adding the ratio of MIC of substance A in combination with its MIC alone, to the ratio of MIC of substance B in combination with its MIC alone. The FIC index is interpreted as follows: FICI ≤0.5 indicates total synergism; 0.5 < FICI ≤0.75 indicates partial synergism; 0.75 < FICI ≤2 indicates no effect; and FICI >2 indicates antagonism [20].

### 2.6 Bacterial cell membrane disruption assay

The crystal violet uptake assay evaluated bacterial cell membrane disruption at sub-minimum inhibitory concentrations (sub-MIC) concentrations. Bacterial suspensions (10^7^ CFU/mL) were prepared in Luria-Bertani (LB) broth, centrifuged at 10,000×*g* for 5 min, washed twice, and resuspended in 50 mM PBS (pH 7.4). Cu-NPs were added at a concentration of MIC/2 and incubated at 37 °C for 30 min, while control samples underwent the same process without Cu-NPs. After another centrifugation, cells were resuspended in PBS with 10 μg/mL crystal violet and incubated at 37 °C for 10 min. Following another centrifugation, the optical density (OD) at 570 nm of the supernatant was measured, with the initial crystal violet OD set at 100 % to determine the uptake percentages. Crystal violet uptake percentages were calculated using the following formula [21]:

$$\frac{OD\;value\;of\;the\;sample}{OD\;value\;of\;crystal\;violet\;solution\;}\times 100$$

### 2.7 Anti-biofilm activity of Cu-NPs

The anti-biofilm activity of Cu-NPs was assessed using the microtiter plate (MtP) assay [22]. Cu-NPs were prepared in MHB at sub-MIC concentrations: MIC/2, MIC/4, MIC/8, and MIC/16. Overnight cultures of *S. aureus*, *B. cereus*, *E*. *coli*, and *P. aeruginosa* were grown in MHB and adjusted to a turbidity of 0.5 McFarland standard, then diluted to 1:100 in fresh MHB to achieve approximately 5 × 10^5^ CFU/mL in each well. In each well, 50 μL of the diluted bacterial suspension was combined with 50 μL of the Cu-NPs solutions at the specified concentrations. A negative control containing the bacterial suspension and MHB without Cu-NPs was also included. The plates were incubated at 37 °C for 18–24 h. After incubation, biofilm formation was quantified using the crystal violet staining method. Planktonic cells were removed, and adherent cells were stained with 200 μL of 0.1 % crystal violet for 20 min. The excess stain was washed off with PBS three times. The stained biofilm cells were solubilized with 100 μL of 95 % ethanol, and OD was measured at 570 nm using a microplate reader (ELx808, BioTek, USA). Each experiment was conducted in triplicate, and results were reported as mean ± standard deviation (SD). Biofilm inhibition was evaluated by comparing Cu-NP-treated samples to the control group.

### 2.8 NorA efflux pump inhibition assay

The study investigated the *S. aureus* SA-1199B strain, which overexpresses the NorA efflux pump, using the ethidium bromide (Et–Br) accumulation assay [23]. Overnight cultures of *S. aureus* SA-1199B were grown in MHB to an optical density of 0.6 at 600 nm, then centrifuged at 5000×*g* for 5 min. The cell pellets were resuspended in 2 mL of phosphate buffer (pH 7), mixed, and transferred to 96-well plates. A saline solution of Et–Br (8 μg/mL) and Cu-NPs at half the minimum inhibitory concentration (MIC/2) was added to the wells. The plates were analyzed using a Corbett Life Science Rotor-Gene 6000 Cycler (Qiagen, Germany) set to an excitation wavelength of 518 nm and an emission wavelength of 605 nm. The fluorescence difference between the Cu-NP-treated samples and controls (containing only Et–Br) showed that Cu-NPs effectively inhibit the efflux of Et–Br.

### 2.9 Anti-swarming motility

This test evaluated the swarming motility of *P. aeruginosa* on plates containing 1 % tryptone, 0.5 % NaCl, 0.5 % glucose, and 0.5 % agar. Two sets of plates were prepared: one with Cu-NPs at MIC/2 concentration and a control set without NPs. After air-drying for 10 min, bacterial cells were inoculated at the center of each plate using a toothpick after drying the surface for 10 min, and swarming activity was observed after a 24-h incubation at 30 °C [22].

### 2.10 Statistically analysis

Statistical analysis was performed using GraphPad Prism (version 6). Each experiment was conducted independently in triplicate and repeated three times for reliability. Data are presented as mean ± SD. An independent two-sample *t*-test was used for comparisons between treated and control groups, while one-way ANOVA with Tukey's post hoc test identified differences among multiple groups. A significance level of p < 0.05 was set, with exact p-values reported for clarity. Normality and homogeneity of variance were confirmed prior to analysis.

## 3 Results

### 3.1 Characterization of synthesized Cu-NPs

#### 3.1.1 UV–vis spectroscopy analysis

**Fig. 2:**
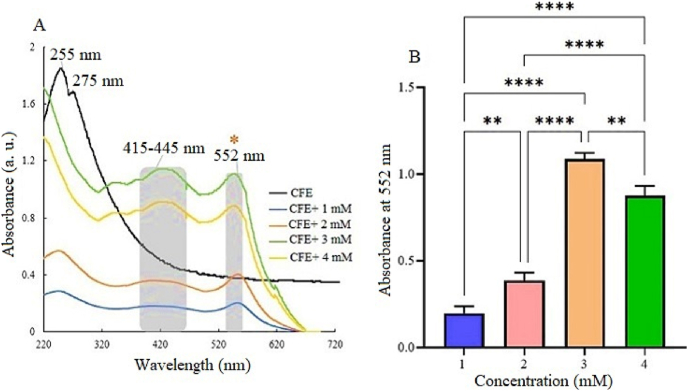
(A) UV–Vis spectra of Cu-NPs synthesized using bacterial supernatant at Cu (NO_3_) _2_·3H_2_O concentrations of 1–4 mM, demonstrating SPR peaks at 415–445 nm and 552 nm. (B) Bar graph of absorbance at 552 nm shows increased nanoparticle formation with higher Cu^2+^ concentrations. Statistical significance: p < 0.01, ∗p < 0.001, ∗∗∗∗p < 0.0001.

Fig. 2 shows the UV–Vis absorption spectra of Cu-NPs synthesized from *Ralstonia* sp. KF264453 bacterial extract, with Cu^2+^ ion concentrations of 1–4 mM (Cu(NO_3_)_2_·3H_2_O). The control sample displayed absorption peaks at 255 and 275 nm, attributed to organic compounds acting as reducing agents. In the presence of copper ions, new peaks appeared at 415–445 nm and 552 nm, indicating surface plasmon resonance (SPR) in the Cu-NPs. A peak at 575 nm suggested the presence of non-oxidized Cu-NPs. The SPR, typically found in the 550–580 nm range for Cu-NPs, increased with Cu^2+^ concentration up to 3 mM, indicating better nanoparticle formation. However, at 4 mM, absorbance slightly decreased, possibly due to aggregation. Statistically significant differences (p < 0.01 and p < 0.0001) suggest that optimal nanoparticle formation occurs at higher Cu^2+^ concentrations.

#### 3.1.2 FESEM/EDX analysis

**Fig. 3:**
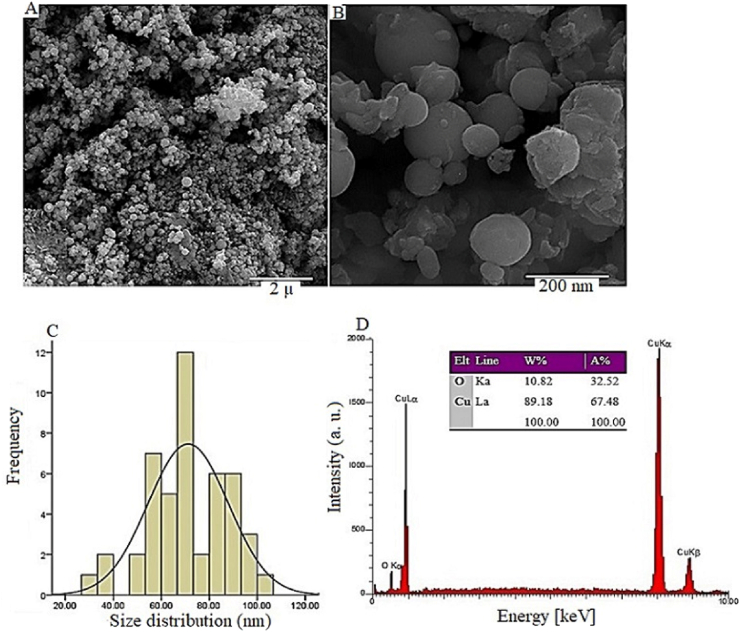
(A, B) FESEM images of biosynthesized Cu-NPs; (C) particle size distribution histogram; (D) EDX spectrum of the NPs.

The FESEM images (Fig. 3A and B) show that the synthesized Cu-NPs are mainly spherical and exhibit some agglomeration, a common trait of biosynthesized NPs stabilized by biomolecules. This morphology, along with a high surface area-to-volume ratio, enhances interactions with bacterial membranes and improves antibacterial effectiveness.The particle size distribution (Figure C) indicates an average size of 69.7 nm, optimal for penetrating bacterial biofilms and ensuring consistent antibacterial activity. EDX analysis (Figure D) confirms the NPs high purity, comprising 89.18 % copper and 10.82 % oxygen, indicating a thin oxide layer that improves stability and interaction with bacterial cells. Overall, the biosynthesis process effectively yields uniform, high-quality NPs.

#### 3.1.3 DLS and zeta potential analysis

**Fig. 4:**
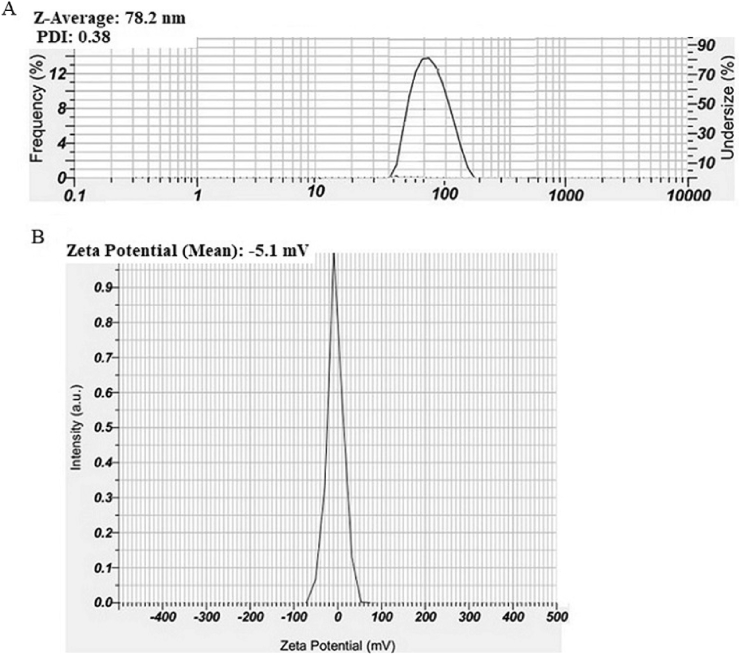
DLS (A) and ZP (B) analyses of Cu-NPs synthesized with the supernatant of *Ralstonia* sp. KF264453.

The DLS analysis (Fig. 4A) reveals a Z-average size of 78.2 nm with a Polydispersity index (PDI) of 0.38, indicating high uniformity and minimal particle aggregation, suitable for antibacterial applications. The Zeta Potential (ZP) measurement (Fig. 4B) of −5.1 mV suggests moderate electrostatic stability for short-term use, with potential for further optimization to improve long-term performance.

#### 3.1.4 FTIR and XRD analysis

**Fig. 5:**
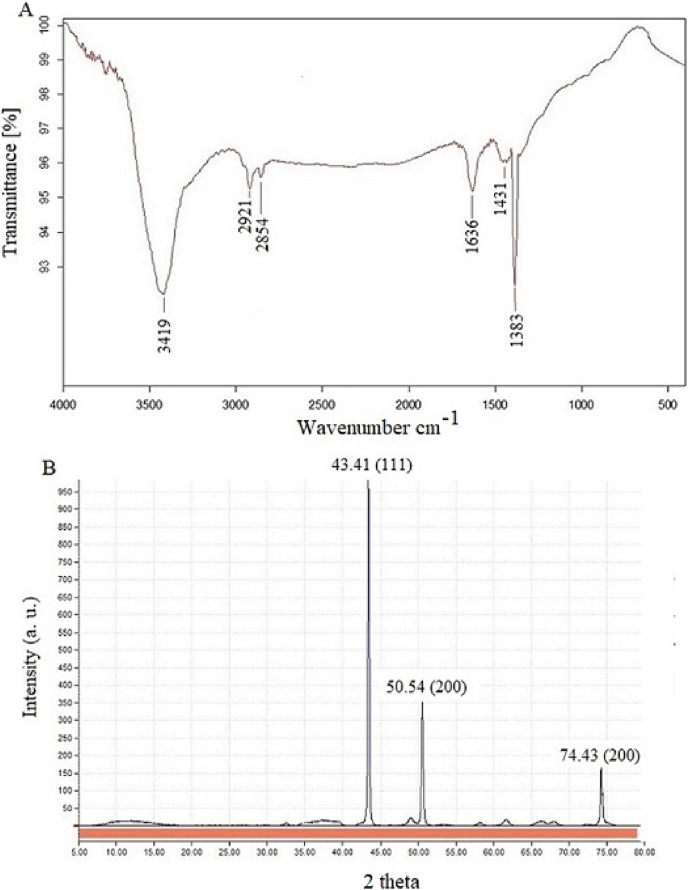
XRD (A) and FT-IR (B) analyses of Cu-NPs from *Ralstonia* sp. KF264453 supernatant.

The FTIR analysis (Fig. 5A) of Cu-NPs highlights the functional groups involved in nanoparticle synthesis and stabilization, revealing significant peaks at 3419 cm^−1^ (O–H stretching of hydroxyl groups), 2921 cm^−1^ and 2854 cm^−1^ (C–H stretching of aliphatic chains), 1636 cm^−1^ (C=O stretching, likely from carbonyl groups), 1431 cm^−1^ (C–H bending in aromatic rings), and 1383 cm^−1^ (C–OH bending vibrations) [24]. The results indicate that biomolecules present in the bacterial extract function as both reducing and capping agents during the nanoparticle synthesis process. The XRD pattern illustrated in Fig. 5B verifies the crystalline structure of the Cu-NPs, displaying diffraction peaks at 2θ values of 43.41°, 50.54°, 74.20°, and 74.43°, which correspond to the (1 1 1), (2 0 0), and (2 2 0) Miller indices. These observed peaks correspond to the reference pattern from JCPDS No. 04–0836, confirming that the Cu-NPs possess a Face-Centered Cubic (FCC) structure [25,26]. The average crystallite size, calculated using Scherrer's equation, is around 45 nm, which suggests that the NPs are at the nanoscale and demonstrate a high degree of crystallinity.

### 3.2 Antibacterial activity of synthesized Cu-NPs

The synthesized NPs demonstrated significant antibacterial activity against all four tested strains, with MIC values of 0.625–5 μg/mL and MBC values of 5–20 μg/mL. Table 1 details the MIC, sub-MIC, and MBC values for the Cu-NPs against *S. aureus*, *B. cereus*, *E. coli*, and *P. aeruginosa*. The NPs showed higher antimicrobial efficacy against Gram-positive bacteria than Gram-negative strains.

**Table 1:** MIC, sub-MIC concentrations, and MBC values for Cu-NPs against the tested bacteria.

Bacteria	MIC (μg/mL)	sub-MIC concentrations (μg/mL)				MBC(μl/ml)
		MIC/2	MIC/4	MIC/8	MIC/16	
*S. aureus*	1.25	0.625	0.312	0.156	0.078	5
*B. cereus*	0.625	0.312	0.156	0.078	0.039	2.5
*E. coli*	2.5	1.25	0.625	0.312	0.156	10
*P. aeruginosa*	5	2.5	1.25	0.625	0.312	20

### 3.3 Synergistic interaction between Cu-NPs and antibiotics

The interaction of Cu-NPs with penicillin was tested against *S. aureus* and *B. cereus*, and with cefixime against *E. coli* and *P. aeruginosa*. Table 2 revealed FICi values below 0.75 for all strains, demonstrating significant total or partial synergy between Cu-NPs and the antibiotics.

**Table 2:** FIC index of Cu-NPs in combination with penicillin and cefixime against pathogenic bacteria.

MICs and FIC _index_^a^	Tested bacteria			
	S. aureus	B. cereus	E. coli	P. aeruginosa
MIC of penicillin	10	5	–	–
MIC of cefixime	–	–	2.5	10
MIC of NPs	1.25	0.625	2.5	5
Combination MIC (antibiotic with NPs)	0.625	0.312	0.625	1.25
FIC _index_	0.562^b^	0.562^b^	0.5^a^	0.375^a^

a (a: total synergism, b: partial synergism).

### 3.4 Bacterial cell membrane disruption

**Fig. 6:**
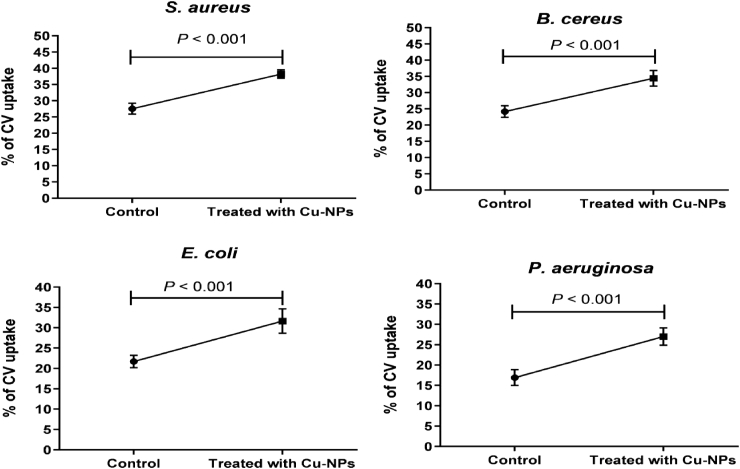
Bacterial cell membrane disruption activity with Cu-NPs at MIC/2 concentration, assessed by crystal violet uptake.

The impact of Cu-NPs at a concentration of MIC/2 on bacterial cell membranes was evaluated using a crystal violet uptake assay. As shown in Fig. 6, the synthesized Cu-NPs significantly enhanced membrane permeability in all four bacterial strains tested-*S. aureus*, *B. cereus*, *E. coli*, and *P. aeruginosa*-with a statistically significant difference (P < 0.001). These findings demonstrate that Cu-NP treatment substantially increased crystal violet uptake compared to the control group, indicating their efficacy in disrupting bacterial cell membranes.

### 3.5 Anti-biofilm activity of Cu-NPs

**Fig. 7:**
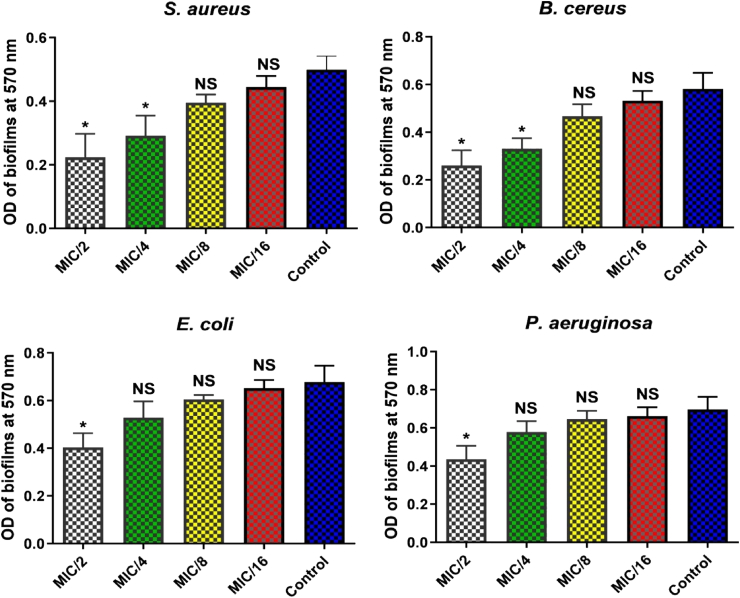
Biofilm inhibition by Cu-NPs at concentrations MIC/2, MIC/4, MIC/8, and MIC/16 against *S. aureus*, *B. cereus*, *E. coli*, and *P. aeruginosa*; (∗: statistically significant, NS: not significant).

The anti-biofilm assays showed that synthesized Cu-NPs effectively inhibited biofilm formation in *S. aureus* and *E. coli* at sub-MIC. Specifically, Cu-NPs significantly reduced biofilm formation in *S. aureus* at MIC/2 and in *E. coli* at both MIC/2 and MIC/4, as illustrated in Fig. 7. For *S. aureus*, the OD of biofilms at 570 nm significantly decreased at MIC/2, while the effect was less pronounced at higher concentrations (MIC/4 and above). *E. coli* exhibited similar results, with notable inhibition at MIC/2 and a maintained but non-significant effect at MIC/4. Among other bacterial strains tested, such as Bacillus cereus and *P*. *aeruginosa*, only MIC/2 of Cu-NPs led to a significant reduction in biofilm formation, with no significant differences at higher concentrations compared to the control. These findings underscore the potential of Cu-NPs as an effective agent against biofilm formation in pathogenic bacteria.

### 3.6 Ethidium bromide efflux pump inhibition

**Fig. 8:**
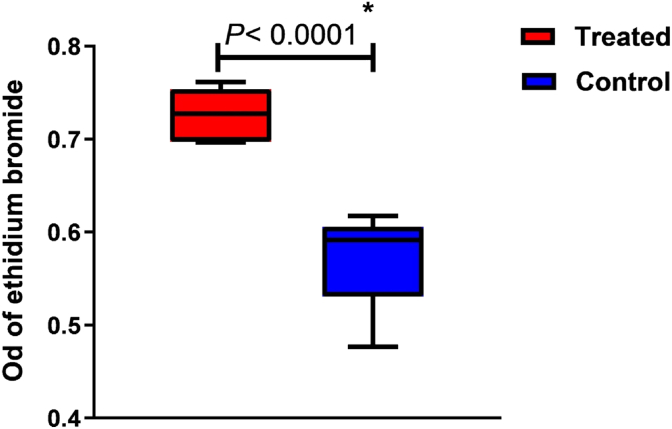
Effect of Cu-NPs on ethidium bromide accumulation; (∗: statistically significant, P < 0.0001).

Fig. 8 demonstrates that Cu-NPs at MIC/2 significantly increased Et–Br accumulation in the *norA*-overexpressing *S. aureus* strain SA-1199B (Fig. 6), strongly indicating inhibition of the NorA pump's activity (P < 0.0001). This enhanced Et–Br accumulation suggests Cu-NPs not only exhibit direct antimicrobial effects but may also boost antibiotic efficacy by disrupting efflux pump function. These findings highlight Cu-NPs as promising candidates for combating antibiotic resistance in pathogenic bacteria.

### 3.7 Anti-swarming motility

**Fig. 9:**
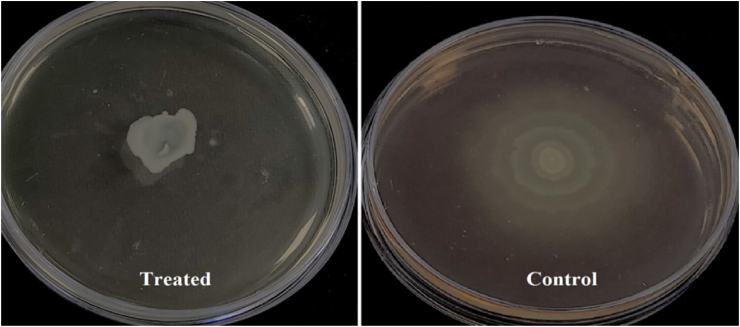
Effect of Cu-NPs on the swimming motility of *P. aeruginosa* at MIC/2 concentration.

We assessed the impact of Cu-NPs on the swarming motility of *P. aeruginosa* at a concentration of MIC/2 using an anti-swarming motility assay. Fig. 9 demonstrates that Cu-NPs significantly inhibited swarming behavior, evident from the reduced spread of bacterial colonies on the treated plate compared to the control. In the control group, colonies exhibited a typical extensive circular growth pattern. In contrast, the treated plate showed minimal bacterial movement and growth, indicating that Cu-NPs effectively restricted swarming motility.

## 4 Discussion

### 4.1 Green synthesis and characterization of Cu-NPs

Copper, an essential trace element historically used for coins and dental materials, is vital for angiogenesis, bone formation, wound healing, and enzyme function [7]. The antibacterial [7,15,27], anticancer [[28], [29], [30]] and antioxidant [31] activities of copper were reported frequently. It supports microbial physiology and acts as a microbicide at high concentrations [32]. Notably, copper is less toxic to mammalian cells than silver and has been recognized for its antimicrobial properties since before antibiotics [33]. Recently, eco-friendly synthesized Cu-NPs and CuO-NPs have gained attention for their safe, cost-effective, and effective antimicrobial applications, particularly against multidrug-resistant (MDR) bacteria [27]. This study reports the first successful synthesis of Cu-NPs using *Ralstonia* sp. KF264453. We characterized their physical properties and evaluated their antibacterial activity, synergistic effects with two common antibiotics, and their effectiveness against biofilm formation and virulence in pathogenic Gram-positive and Gram-negative bacteria. UV–Vis spectroscopy revealed Cu-NPs with an absorption peak at 552 nm. FESEM and DLS analyses showed the NPs were primarily spherical, averaging 69.2 nm in diameter and having a Z-Average size of 78.2 nm. A zeta potential of −5.1 mV indicates moderate stability. FT-IR analysis revealed functional groups associated with the Cu-NPs, suggesting potential interactions with bacterial cells. These findings highlight the potential of Cu-NPs from *Ralstonia* sp. KF264453 as effective antimicrobial agents, particularly against MDR bacteria, offering an alternative to conventional antibiotics. Compared to earlier studies, our analysis revealed differences in the characteristics of synthesized Cu-NPs, such as size, shape, size distribution, and surface charge. Other research indicated that Cu-NP sizes range from 5 to 150 nm [27], with discrepancies likely due to factors like the microorganism used, production methods, and environmental conditions during synthesis. Such differences can significantly affect the biological potential of the NPs [34]. The green synthesis of Cu-NPs offers advantages, including environmental friendliness by using natural resources like plant extracts and microorganisms, which reduces toxic chemical use and hazardous waste [9]. It is also cost-effective, requiring less energy and simpler equipment than conventional methods. Phytochemicals and enzymes enhance nanoparticle stabilization, ensuring quality and functionality [9,35]. Additionally, green synthesis allows for controlled size and morphology of Cu-NPs, improving their biological and antimicrobial activities, thus presenting a sustainable alternative for various nanotechnology applications [36]. Surface functionalization of NPs is crucial for enhancing stability, biocompatibility, and functionality in biomedical applications [37]. In green-synthesized Cu-NPs, biomolecules like proteins, enzymes, and phytochemicals from the synthesis medium stabilize the NPs, reduce aggregation, and improve biological interactions, thereby boosting their antimicrobial and biological properties. These features are vital for various bio-applications. While this study emphasizes natural functionalization, future research could explore targeted methods to optimize Cu-NPs for specific clinical uses.

### 4.2 Antibacterial, anti-biofilm, and synergistic effects of Cu-NPs

Following the synthesis and characterization of Cu-NPs, we assessed their antibacterial activity against four pathogenic bacteria. The results showed significant antibacterial effects, with MIC values ranging from 0.625 to 5 μg/mL and MBC values between 2.5 and 20 μg/mL. Moreover, Cu-NPs markedly improved the efficacy of penicillin against *S. aureus* and *B. cereus*, and cefixime against *E. coli* and *P. aeruginosa*, yielding FICi values below 0.75. Cu-NPs and Cu-functionalized nanomaterials exhibit antibacterial properties against a wide range of microorganisms, including Gram-positive and Gram-negative bacteria and fungi [7,[38], [39], [40]]. Zhao et al. found that Cu-NPs synthesized with *Allium eriophyllum* leaf extract effectively targeted multiple bacterial species, including *S. aureus*, *B. subtilis*, *E. coli*, and *P. aeruginosa*, as well as various fungi like *Candida guilliermondii*, *C. krusei*, *C. glabrata*, and *C. albicans* [41]. Researchers used a cell-free extract (CFE) from *Stenotrophomonas* sp. BS95 to synthesize CuO-NPs, which exhibited antibacterial activity with MIC between 62.5 and 1000 μg/mL against Gram-negative and Gram-positive bacteria. These NPs also showed notable antioxidant and anticancer properties [42]. This study, along with similar research, shows that Cu-NPs and CuO-NPs have antimicrobial properties, with MIC values ranging from under 1 to about 1000 μg/mL. However, assigning a specific MIC value for Cu-NPs is challenging due to variability across studies, highlighting the complexity of nanoparticle behavior influenced by size, surface charge, and functionalization. Standardizing experimental conditions and nanoparticle characteristics is essential for achieving consistent results in future research [[40], [41], [42]]. We also reported the synergistic activity of Cu-NPs with penicillin and cefixime, which may help restore susceptibility in antibiotic-resistant bacteria [43]. These compounds can disrupt bacterial cell membranes or walls, facilitating antibiotic penetration [44]. Another mechanism involves inhibiting bacterial efflux pumps, suggesting that combination therapy could effectively manage emerging resistant strains [43]. Given their unique properties and small size, NPs are promising candidates for use alongside antibiotics in treating infectious diseases. Previous research has frequently highlighted this potential, with similar findings reported for various metal NPs, including silver and iron NPs [45]. To investigate the antimicrobial mechanism of Cu-NPs against tested pathogens, we evaluated their effect on bacterial cell membrane disruption using the CV uptake assay. Results indicated that Cu-NPs significantly increased CV uptake in all tested bacteria, suggesting damage to the bacterial cell membrane. This finding implies that the observed synergistic interactions may enhance antibiotic penetration by increasing cell membrane permeability following Cu-NP treatment [46]. Metal NPs have shown significant potential in damaging bacterial cell membranes, contributing to their antibacterial properties [5,46]. Silver NPs (Ag-NPs) can penetrate the bacterial cell wall and form pores on the membrane surface, leading to free radical formation that further damages the cell membrane [47]. Similarly, Gold NPs (Au-NPs) induce antibacterial activity by causing membrane damage, DNA damage, and disruption of the electron transfer chain [48]. Positively charged metal-based NPs form strong electrostatic interactions with negatively charged bacterial membranes, leading to cell wall disruption and increased permeability [46]. The size of NPs is crucial for their ability to disrupt bacterial membranes. Typically ranging from 2 to 10 nm, smaller NPs exhibit stronger antibacterial effects due to their larger surface area for contact with bacterial cells [49].

Researchers increasingly focus on compounds with antivirulence characteristics, as they typically do not kill bacteria directly, reducing the likelihood of resistance development [6,23,50]. A recent study investigated the antivirulence properties of Cu-NPs against four bacterial strains, focusing on biofilm formation inhibition, NorA efflux pump inhibition in *S. aureus*, and bacterial motility inhibition in *P. aeruginosa*. Results showed that at sub-MIC concentrations, Cu-NPs effectively inhibited biofilm formation, affected efflux pump activity, and reduced bacterial motility. Bacterial biofilm formation is a major public health concern, significantly enhancing resistance to antimicrobial agents and host immune responses—sometimes by as much as 1000 times. Identifying compounds with effective anti-biofilm properties is crucial [51,52]. NPs have demonstrated the ability to disrupt or inhibit bacterial biofilms through various mechanisms, including interfering with bacterial adhesion, preventing biofilm maturation, and altering gene expression patterns associated with biofilm development. For instance, NPs can influence quorum sensing (QS) system gene expression to inhibit biofilm formation [5,53]. Additionally, NPs inhibit efflux pumps, mechanisms bacteria use to expel antimicrobial agents, enhancing traditional antibiotic efficacy against resistant strains like methicillin-resistant *S. aureus* (MRSA) [5,54]. NPs, particularly silver ions and biogenic Ag-NPs, effectively reduce the motility of *E. coli* and *P. aeruginosa*, limiting their ability to colonize surfaces and form biofilms [55]. Targeting antivirulence mechanisms offers numerous advantages, reducing selective pressure that typically leads to resistance development. This approach holds promise for combating existing antibiotic-resistant strains and opens avenues for combination therapies that enhance overall treatment effectiveness.

## 5 Conclusion

This study investigated the green synthesis of Cu-NPs with *Ralstonia* sp. KF264453 and evaluated their antibacterial, anti-biofilm, and antivirulence activities. The eco-friendly synthesis approach employed in this research demonstrated both sustainability and reproducibility, offering an environmentally responsible alternative for nanoparticle production. UV–Vis spectroscopy, FESEM, and DLS analysis characterized Cu-NPs as spherical with an average size of 69.2 nm. These NPs demonstrated strong antibacterial effects, particularly when combined with penicillin and cefixime, effectively inhibiting biofilm formation, efflux pump activity, and bacterial motility. These findings highlight Cu-NPs as a promising therapeutic option and a complement to traditional antibiotics for resistant bacterial strains. The study suggests their effectiveness against MDR bacteria by targeting key virulence factors. Additionally, it paves the way for further research into their mechanisms of action, antioxidant and anti-inflammatory properties, and clinical biocompatibility. Ultimately, Cu-NPs could be crucial in addressing bacterial resistance and the growing decline in antibiotic efficacy.

## CRediT authorship contribution statement

**Narges Vakili:** Methodology. **Morahem Ashengroph:** Writing – review & editing, Validation, Project administration. **Aram Sharifi:** Writing – original draft, Software, Methodology. **Musa Moetasam Zorab:** Methodology, Investigation.

## Ethics statement

Not applicable.

## Data Availability

The data that support the findings of this study are available from the corresponding authors as the corresponding author, upon reasonable request.

## Study limitations

This study is limited by the lack of detailed elucidation of Cu-NPs' mechanisms of action and their antioxidant, anti-inflammatory, and biocompatibility properties. Furthermore, *in vivo* studies and clinical evaluations were not conducted, leaving these areas for future research.

## Funding

The authors are grateful to the research Council of the 10.13039/501100008973University of Kurdistan for their financial support under Grant Agreement Number 00/9/34,027/2021.

## Declaration of competing interest

The authors declare the following financial interests/personal relationships which may be considered as potential competing interests: The authors declare that they have no known competing financial interests or personal relationships that could have appeared to influence the work reported in this paper.

## Data Availability

Data will be made available on request.
